# Genetic diversity within and among Atlantic cod (*Gadus morhua*) farmed in marine cages: a proof-of-concept study for the identification of escapees

**DOI:** 10.1111/j.1365-2052.2010.02025.x

**Published:** 2010-10

**Authors:** K A Glover, G Dahle, J I Westgaard, T Johansen, H Knutsen, K E Jørstad

**Affiliations:** * Institute of Marine ResearchPO Box 1870, Nordnes. N-5817 Bergen, Norway; † Institute of Marine ResearchPO Box 6404, NO-9294 Tromsø, Norway; ‡ Institute of Marine ResearchNye Flødevigveien 20, N-4817 HIS, Norway

**Keywords:** aquaculture, assignment, escapee, microsatellite, *Pan I*, traceability

## Abstract

This study presents a molecular genetic characterization of Atlantic cod reared in commercial marine farms. Samples consisted of approximately 47 fish collected from nine cages located on four farms throughout Norway. In addition, 28 farmed escapees were recaptured in the sea (443 fish in total). Nine microsatellite loci and the *Pan I* gene were analysed, revealing a total of 181 alleles. Each sample contained 43–63% of total allelic variation. Comparing variation with published data for wild cod indicates that lower genetic variation exists within single cages than in wild populations. Significant linkage disequilibrium was observed amongst pairs of loci in all samples, suggesting a low number of contributing parental fish. Global *F*_ST_ was 0.049, and the highest pairwise *F*_ST_ value (pooled loci) was 0.085. For single loci, the *Pan I* gene was the most diagnostic, displaying a global *F*_ST_ of 0.203. Simulations amongst the samples collected on farms revealed an overall correct self-assignment percentage of 75%, demonstrating a high probability of identifying individuals to their farm of origin. Identification of the 28 escapees revealed a single cage as the most likely source of origin for half of the escapees, whilst the remaining fish were assigned to a mixture of samples, suggesting more than one source of escapees.

## Introduction

The Atlantic cod (*Gadus morhua* L.) represents one of the most economically important fish in the north Atlantic. However, serious decline in abundance has been observed in many coastal stocks, a situation which has stimulated interest for cod farming. In Norway, which is one of the primary producing countries, current aquaculture production of cod is approximately 10 000 tonnes/year. This industry has considerable potential to expand, and the Norwegian government has issued sufficient farming licences to enable a commercial production of approximately 300 000 tonnes/year.

Heritability of commercially important production traits in cod has been studied (e.g. Gjerde *et al.* 2004; Kolstad *et al.* 2006), and breeding programmes have been established. Despite this, the industry may be regarded as being in its infancy, and some production is still based upon the spawning of wild captured adults. Larvae and fry production include the application of both extensive and intensive technology, including mass-spawning tanks. Breeding success is often skewed in mass-spawning tanks (Herlin *et al.* 2008 and references therein), potentially leading to reduced genetic variation.

Cod have been a subject to a large number of molecular genetic studies to delineate population structure (e.g. Frydenberg *et al.* 1965; Pampoulie *et al.* 2006a; Jorde *et al.* 2007; O′Leary *et al.* 2007; Westgaard & Fevolden 2007). However, with the exception of broodstock screening in connection with stock enhancement programmes (Jørstad 1986; Jørstad *et al.*1994), and analysis of experimental farmed strains (Pampoulie *et al.* 2006b), there is no molecular genetic characterization of cod in commercial production. This contrasts with the situation in Atlantic salmon (*Salmo salar* L.), where a number of studies have been conducted, generally reporting reduced genetic variation and increased genetic differentiation in and among farmed strains compared with wild fish populations (e.g. Mjølnerød *et al.* 1997; Norris *et al.* 1999; Skaala *et al.* 2004).

A major challenge for aquaculture is containment. In Norway, the reported numbers of farmed escaped cod have ranged from 20 000 to 290 000 yearly in the period 2004–2008, although the real figure is probably higher because of under-reporting (Baarøy *et al.* 2004). There is universal concern over the potential for negative genetic interactions between farmed escaped fish and their wild counterparts (Genimpact 2006). Recently, Glover *et al.* (2008) used genetic assignment to successfully identify the farm of origin for escaped Atlantic salmon recaptured in a Norwegian fjord. These authors have developed the approach for identification of escapees in further studies (Glover 2008; Glover *et al.* 2009), which involves matching the multi-locus genetic profile of individual escapees with groups of fish sampled in commercial production cages. However, sufficient among-farm genetic variation is a pre-requisite for the robust identification of escapees to their farm of origin, and this needs to be quantified to enable evaluating whether it is possible to apply a similar technique to other aquaculture species, such as cod.

This study was designed to investigate the level of genetic variation observed in groups of cod reared in commercial production cages, and to quantify the level of genetic differentiation amongst cages and farms. This was conducted with the primary intention of evaluating the potential of using genetic assignment to identify the cage and farm of origin for escapees, and also to perform a genetic characterization of farmed Atlantic cod in Norway.

## Materials and methods

### Biological samples

A total of nine samples were collected from four commercial grow-out farms in the period January–March 2009 (Table 1). Each sample consisted of approximately 50 fin clips taken from fish reared in a single marine cage. For farms that reared more than one strain of cod, i.e. fish of a different genetic background or delivered by more than one juvenile producer (i.e. a hatchery producer of small fish ready for grow-out), a cage representing each of these genetic groups was sampled. These are henceforth referred to as the baseline samples and represent potential sources of fish escape to be considered in the assignment simulations conducted herein. In addition, 28 farmed escaped cod were captured by a commercial gill-net fisherman close to farm E in March 2009. Tissue samples were secured from these fish.

**Table 1:** Origin and background of nine samples of caged Atlantic cod collected on four farms (E, G, H and S), and a group of escapees (RF) recaptured in the vicinity of farm E

Sample	Fry producer	Genetic strain	Mean weight (kg)	Sampling date	Date placed in cage
E1	A	1	1.1	05.03.09	27.08.08
E5	A	1	2.2	05.03.09	13.05.08
E2	B	2	2.4	05.03.09	13.05.08
E9	B	2	1.4	05.03.09	02.11.08
G4	C	3	1.5	28.03.09	30.06.08
G3	D	2	1.3	28.03.09	14.10.07
G6	E	4	1.9	28.03.09	05.05.07
H4	A	1	1.5	25.03.09	10.02.08
S	F	5	2.2	25.03.09	15.10.07
RF	?	?	–	12.03.09	?

### Genotyping

DNA was isolated in 96 well format using the Qiagen DNAeasy extraction kit at the Institute of Marine Research (IMR). In addition to the *Pan I* locus (Fevolden & Pogson 1997), nine microsatellite loci were analysed: *Gmo 3*, *Gmo 8*, *Gmo 34*, *Gmo 35* and *Gmo 37* (Miller *et al.* 2000); *Gmo 2* and *Gmo132* (Brooker *et al.* 1994); and *Tch 11* and *Tch 13* (O'Reilly *et al.* 2000). The protocol of Westgaard & Fevolden (2007) was slightly modified to allow for a 2.5 μl reaction volume in the PCR. The amplified alleles were separated using an ABI 3130 XL sequence analyser (Applied Biosystems) and scored with the software Genemapper 4.0 (Applied Biosystems). The *Pan I* locus was genotyped according to Stenvik *et al.* (2006) using one unlabelled forward primer and two different reverse primers; one *Pan I*^A^ specific primer labelled with 6-FAM, and one *Pan I*^B^ specific primer labelled with PET. The PCR products were run on an ABI3730 sequence analyser and scored with GeneMapper v4.0.

### Statistical analysis

The samples were characterized by a range of standard population genetic tests and parameters conducted in various programmes. The program msa (Dieringer & Schlötterer 2003) was used to compute summary and *F*-statistics. Genepop V3.3 (Raymond & Rousset 1995) was used to test for deviation from Hardy–Weinberg equilibrium (HWE), and to test for linkage disequilibrium (LD), both using a Fisher's exact test (demorization 10 000; 100 batches; 5000 iterations). LD was tested for all pairs of loci in all samples. fstat (Goudet 2001) was used to compute allelic richness. A matrix of pairwise *F*_ST_ values was used in the program mega (Tamura *et al.* 2007) to produce a phylogenetic tree using the UPGMA method (Sneath & Sokal 1973). The tree was linearized, assuming equal evolutionary rates in all lineages, according to Takezaki *et al.* (2004).

Bayesian clustering analysis implemented in STRUCTURE 2.2 (Pritchard *et al.* 2000; Falush *et al.* 2003) was used for estimating the number of populations/groups (*k*) represented by all the sampled individuals (including escapees) and for assigning individuals to these groups without using prior information about their origin. Runs were conducted at *k*= 1–10, each with five iterations. Correlated allele frequencies and an admixture model were assumed. Each run consisted of a burn-in of 50 000 MCMC steps, followed by 200 000 steps. STRUCTURE was also used to assign escapees to the cage-samples using prior information about sample of origin for the individuals sampled in cages; no prior was used for the escapees (*k* set at 9).

GeneClass2 (Piry *et al.* 2004) was used to perform self-assignment simulations with the samples taken from cages using the leave one out procedure and the Rannala & Mountain (1997) method of computation. This program was also used to perform direct assignment of the escapees and exclusion (*α*= 0.05) with a variety of methods (see Results). Although a range of assignment programs are available (see Hansen *et al.* 2001; Manel *et al.* 2005), GeneClass2 was used for assignment in this study because it permits the calculation of exclusion (i.e. rejection of unknown individuals from baseline samples at different significance levels), which is important in a forensic application. This is also important to counteract the possibility of false assignment in the case of a potentially incomplete baseline.

## Results

### Genetic variation within samples

A total of 181 alleles were observed in the entire data set consisting of 443 fish genotyped at ten loci (Table 2). *GMO 8* (41 alleles) and *GMO 132* (30 alleles) represented the most polymorphic loci, whilst the *Pan I* locus (2 alleles) was the least polymorphic. Individual samples displayed 78–114 alleles across loci, equating to 43–63% of the variation observed in the entire data set.

**Table 2:** Genetic variation observed in cod sampled from nine cages on commercial farms (G3-S), and a group of farmed escapees (RF), genotyped at nine microsatellite loci and the *Pan I* gene

		Locus										Summary			
		
Sample	*N*	*GMO35*	*GMO37*	*GMO8*	*TCH11*	*GMO132*	*GMO2*	*GMO3*	*GMO34*	*TCH13*	*Pan I*	*A*_T_	*A*_M_	*A*_R_	Gene diversity
G3	47	8	7	24	17	21	10^1^	3	6^2^	16	2	114	11.4	91.7	0.67
G4	47	8	8	16^1^	16^2^	15	11^1^	3	4	16^2^	1	98	9.8	78.1	0.64
G6	47	7	9^2^	16^1^	12	11	6	5^2^	5^2^	13	2	86	8.6	67.0	0.65
E1	47	7	10	17	16	22	11	4	4^1^	13^1^	1	105	10.5	81.7	0.65
E2	39	8	7	20	15	19	11	4	6^1^	16	1	107	10.7	89.7	0.68
E5	47	7	10	21	14	14	8^2^	2	6	17^1^	2	101	10.1	82.4	0.66
E9	47	6	9^1^	11^1^	11^1^	10^1^	6	2	7	14	2	78	7.8	62.7	0.64
H4	47	6	8^1^	16^2^	10^2^	11^2^	5	3	5	12	2	78	7.8	63.3	0.64
S	47	8	9	15	17	18	7	3	5	15	2	99	9.9	78.7	0.67
RF	28	9	NG	16^2^	14	14^1^	10	3^1^	5	16	2	89	9.9	87.3	0.66
Total	443	11	14	41	20	30	17	8	8	30	2	181	18.1		0.67
Global *F*_ST_		0.039	0.046	0.046	0.045	0.035	0.070	0.030	0.023	0.037	0.204				
Gene diversity		0.80	0.81	0.88	0.89	0.89	0.76	0.16	0.44	0.88	0.20				

*N*, number of individuals genotyped per sample; *A*_T_, total number of alleles; *A*_M_, mean number of alleles; *A*_R_, allelic richness; NG, locus not genotyped for sample.

1 Significant deviation from Hardy–Weinberg equilibrium (HWE) (*α*= 0.05)

2 Significant deviation from HWE following correction (*α*= 0.005).

From 99 tests, 26 of the population by locus combinations deviated from HWE (*α*= 0.05) (Table 2). Deviations were mostly associated with positive *F*_is_ values (data not presented). Following adjustment for ten tests per sample (new *α*= 0.005), the total number of significant departures dropped to 11, and these were distributed unevenly amongst the samples, with G6 and H4 each displaying three departures (Table 2). However, none of the loci were implicated in more than two significant departures from HWE (post-correction). A total of 123 locus pairs displayed significant LD of 414 tests which could be computed (a total of 450 pairwise tests could be computed but because of some monomorphic loci in some samples only 414 tests were computed). Following sequential Bonferroni correction, 30 of these remained significant. LD was observed for a variety of different locus pairs; however, distribution of LD amongst samples was less even, ranging from none in the sample of escapees (RF) to 9 significant pairs in one of the cage-samples (G4).

### Genetic variation amongst samples

Global *F*_ST_ was computed at 0.049 (0.043 excluding *Pan I*). For individual microsatellite loci, global *F*_ST_ ranged from a low of 0.023 for *GMO 34*, to 0.07 for *GMO 2*. In contrast, the *Pan I* locus displayed greater among-sample variation, with a global *F*_ST_ of 0.204, and a highest pairwise *F*_ST_ of 0.45. The high *F*_ST_ values displayed by *Pan I* reflect the fact that it was monomorphic in three of the samples, but displayed *Pan I*^B^ allele frequencies of 0.46, 0.37, 0.24 and 0.16 in samples S, G6, E9 and RF respectively.

Pooling loci, highly variable *F*_ST_ values were observed pairwise between samples, ranging from a low of 0.008 between G3 and E2, to 0.085 between E1 and G6. Only six of 46 pairwise *F*_ST_ values were below 0.02. Significant genetic differentiation was also observed between cages located on the same farm. For example, samples E2 and E9 displayed a pairwise *F*_ST_ value of 0.069 across all loci.

**Figure 1:**
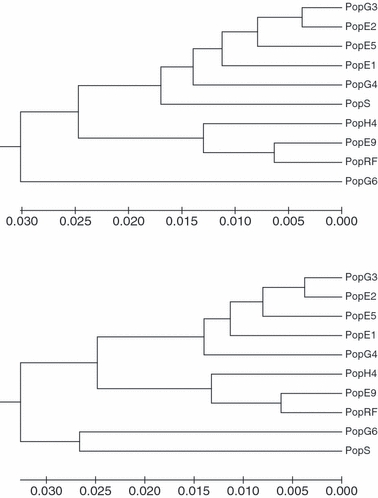
*F*_ST_-based UPGMA diagrams illustrating genetic relationships amongst nine samples of cod taken from marine cages and a group of 28 escapees. Data are based upon nine microsatellite loci (top), or nine microsatellite loci and the *Pan I* gene (bottom).

**Figure 2:**
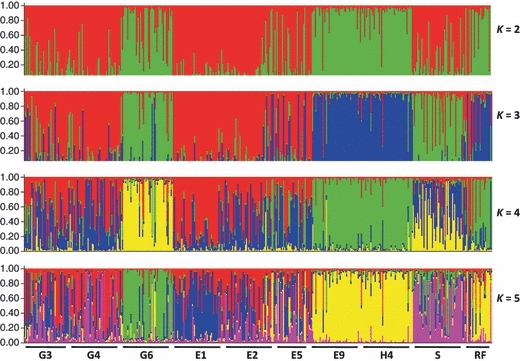
Bayesian clustering of cod sampled from nine marine cages and 28 recaptured escapees. Each vertical bar denotes an individual, whilst colours denote inferred clusters. Note that colours are not universal between *k*= 2 and 5*.*

Three major genetic clusters were identified; however, sample S changed cluster when *Pan I* was included (Fig. 1). This is likely to be a result of its exhibiting a very high frequency of the *Pan I*^B^ allele. In both UPGMA diagrams, the sample consisting of 28 escapees was located in a cluster with E9 and H4. Bayesian clustering of the data revealed significant structure (Fig. 2), which displayed concordance with the three major lineages identified by the UPGMA diagram that included data from all loci (Fig. 1). Increasing from *k* 3 to *k* 5 led to an increase in noise rather than distinct structure; however, the sample of escapees (RF) remained clustered with samples E9 and H4 at all *k* examined.

### Self-assignment simulations

An overall correct self-assignment percentage of 75% was observed amongst the nine samples collected on farms. This remained almost unchanged when the locus *Pan I* was excluded from this analysis (74%), and only minor differences in patterns of incorrect assignment were observed (data not shown). Using a genetic distance-based method, *D*_A_ (Nei *et al.* 1983), and all loci, overall correct self-assignment was also high (73%).

Correct self-assignment ranged from 51 to 89% for individual samples, and the pattern of incorrect assignment varied (Table 3). For example, sample H4 only incorrectly assigned to samples E9 and S, whereas sample E5 was assigned incorrectly to all but two of the samples. Incorrect assignments amongst samples tended to reflect genetic similarity. For example, samples G3 and E2 displayed the greatest similarity of any pairwise comparison (Fig. 2), and clearly incorrect assignment was greatest between these two samples.

**Table 3:** A matrix of self-assignment amongst nine cage-samples of cod using the program GeneClass2. Numbers in bold represent individuals correctly assigned to sample. Overall self-assignment = 75%

	G3	G4	G6	E1	E2	E5	E9	H4	S	*N*	% correct
G3	**24**	6	1		11	1			4	47	51
G4	1	**35**		2	8				1	47	74
G6	2		**42**	1			1		1	47	89
E1	4			**36**	5	2				47	77
E2	7	2		5	**21**	4				39	54
E5	1	1		2	3	**34**	1	5		47	72
E9		2					**42**	3		47	89
H4							4	**41**	2	47	87
S	3	2	3		1			1	**37**	47	79

*N*, number of individuals genotyped per sample.

### Assignment of escapees

All genetic assignment methods implemented identified sample E9 as the most likely origin for 13–15 of the 28 escapees (Table 4). The remaining escapees were directly assigned to a mixture of the baseline samples, with a maximum of four escapees being directly assigned to any one alternative sample.

**Table 4:** Assignment of 28 farmed escaped cod to nine samples from caged cod using different statistical methods implemented in the programs GeneClass2 and STRUCTURE

	Baseline sample								
	
Statistic	G3	G4	G6	E1	E2	E5	E9	H4	S
Direct assignment									
STRUCTURE	2	1	0	0	3	3	15	3	1
Geneclass2^1^ all loci	3	2	0	0	2	4	13	3	1
Geneclass2^1^ no *Pan* I	2	1	0	0	3	3	14	3	2
Geneclass2^2^	1	2	0	0	2	2	14	5	2
Exclusion^3^ (α = 0.05)									
Geneclass2^1^	16	4	3	4	14	12	14	8	3
GeneClass2^2^	20	12	7	11	17	19	19	16	10

1 Rannala & Mountain (1997).

2 Nei *et al.*'s (1983)*D*_A_.

3 A total of 4 and 3 cod were excluded from all samples using methods (1) and (2) respectively.

At the chosen level of probability (*α*= 0.05), samples G3, E2, E5 and E9 could not be excluded as potential sources for 12–16 of the escapees, whilst samples G4, G6, E1, H4 and S could be excluded as potential sources for 20–25 of the 28 escapees. At the same time, 3–4 of the escapees (depending upon method) were excluded from all baseline samples at this probability.

## Discussion

This study has demonstrated that highly significant genetic differentiation exists among groups of cod reared in production cages, both within and between farms. These differences in allele frequencies are driven by a mixture of genetic drift and founder effects acting both within and amongst the commercial strains. Through a combination of genetic analyses, in addition to real-life assignment of 28 unknown escapees, it has been shown that genetic assignment may be used to identify the source of escaped farmed cod, as has previously been described for Atlantic salmon (Glover *et al.* 2008, 2009) and rainbow trout (Glover 2008). Consequently, this proof-of-concept study provides management authorities and commercial producers with an identification tool that will enable greater control over management practices in the industry.

In this study, pairwise *F*_ST_ values were as high as 0.085, and the majority were over 0.02. Even when the highly informative *Pan I* locus was excluded, pairwise *F*_ST_ values as high as 0.075 were observed. The *F*_ST_ values reported in this study are high compared with samples collected from wild cod within regions. For example, in a study of wild cod from southern coast of Norway, Knutsen *et al.* (2003) obtained a global *F*_ST_ of 0.0023, with maximum of 0.0051 for a single locus. Pampoulie *et al.* (2006a) observed a mean *F*_ST_ of 0.003 amongst cod sampled in Iceland, and Ruzzante *et al.* (2001) observed *F*_ST_ values of 0.0039 to 0.0053 in cod sampled at Newfoundland. However, over larger geographical distances, genetic differentiation is more similar to the levels observed here. For example, Nielsen *et al.* (2009) reported a single pairwise value of 0.062 between a sample in the North sea and Northeast arctic cod, and O′Leary *et al.* (2007) reported a pairwise *F*_ST_ value of 0.11 amongst samples from the Scotian shelf and the Baltic sea.

In a study of Northeast Arctic and Norwegian coastal cod on the Lofoten spawning grounds in northern Norway, Wennevik *et al.* (2008) reported numbers of alleles observed per sample for six microsatellite markers overlapping with those used in this study. Although sample sizes were smaller in this study, tentative comparison of allelic variation at mutual loci suggests that the level of genetic variation observed in a single production cage is less than a sample of spawning cod taken from the wild. To illustrate, Wennevik *et al.* (2008) reported the following range in number of alleles per sample across 12 samples, and total number of alleles in the entire data set (in brackets) for the following loci: *GMO 2*= 10–17 (21), *GMO 3*= 3–8 (10), *GMO 132*= 8–22 (33), *TCH 11*= 17–23 (28), *GMO 35*= 7–10 (12), *GMO 34*= 3–8 (8). Both ranges and total numbers of alleles were lower for all of these markers in this study (Table 2). In addition, a large number of pairs of loci were found to display significant linkage disequilibrium here, although this has not been observed for these loci in wild populations (Westgaard & Fevolden 2007). Taken together, these data indicate that a low number of parental fish contributed to the groups of cod reared in the cages sampled in this study. Significant linkage disequilibrium is routinely observed in cage-samples of Atlantic salmon (Glover K. A., personal observation), and this has been reported in hatchery and commercial strains of Atlantic salmon (Withler *et al.* 2005; Innes & Elliot 2006). Genetic drift is higher when few parents contribute to each generation. This elevates the levels of *F*_ST_ between domesticated populations (as reported in this study), thus increasing the chance of assigning escapees to the farm of origin.

Loss of genetic variation in farmed strains compared with their wild counterparts has been documented for the Atlantic salmon (Mjølnerød *et al.* 1997; Norris *et al.* 1999; Skaala *et al.* 2004), in experimental farmed cod strains (Pampoulie *et al.* 2006b), and other marine species (Tanaguchi 2004; Bert *et al.* 2007). Here, whilst reduced genetic variation compared with wild populations was detected, none of the samples displayed exceptionally low levels of genetic variation. This is surprising, as cod are often mass-spawned in large tanks, which tends to result in highly skewed recruitment (e.g. Herlin *et al.* 2008). No clear evidence suggesting such an effect was observed in this study. However, the degree to which any such skewed mass-spawning contribution may have been counteracted, by juvenile producers mixing cod from a number of mass-spawning tanks, remains unclear.

It was not the specific intention of this study to identify markers that would distinguish wild and farmed cod. The principle of tracing escapees back to cage and farm of origin circumvents this particular challenge by using morphological characteristics for identification. Nevertheless, several of the samples analysed in this study contained moderate to high frequencies of the *Pan I*^B^ allele, which is rare in Norwegian coastal cod populations (Fevolden & Pogson 1997; Sarvas & Fevolden 2005). Therefore, where cod strains displaying a high frequency of this allele are farmed in regions where this allele is rare in wild fish, it may serve as a ‘diagnostic’ genetic marker. Farmed cod may be able to impact on wild populations, even without escaping, by spawning in net pens (Jørstad *et al.* 2008). Therefore, regulatory authorities should evaluate the merits of selective breeding programmes for the *Pan I*^B^ allele, or alternatively, genetic incorporation of a rare enzyme allele (Jørstad *et al.* 1991) into farmed cod strains, to actively monitor escapement and potential impacts on wild populations.

In this study, sample E9 was identified as the most likely source of origin for approximately half of the 28 escapees. However, our analyses suggest more than one source for the escapees, which is supported by the fact that sample RF displayed the second highest allelic richness, much higher than sample E9, where approximately half of them were assigned. Only a small fraction of incorrect assignment from E9 to H4 was expected (Table 3), and, consequently, assignments of escapees to samples E2 and E5 indicate that fish have also escaped from these cages. None of the ‘alternative’ baseline samples (G3–G6, H4 or S) were considered as ‘real’ potential sources of the escapees as a result of the fact that they were not located in the same region as farm E. However, it is possible that these fish were from another cod farm located closer to farm E that was not included in this study.

In conclusion, this proof-of-concept study has demonstrated that cod from different cages and/or farms may be genetically divergent, to a degree that enables the identification of escapees back to source without prior knowledge of the pedigree. Atlantic cod aquaculture is a growing industry, and it is suggested that continued genetic monitoring of the domestication process will be important to produce well-informed decisions that will enable the sustainable co-existence of wild and cultured populations.
